# Informing estimates of probability of *Clostridioides difficile* infection for testing and treatment: expert consensus from a modified-Delphi procedure

**DOI:** 10.1017/ash.2024.387

**Published:** 2024-10-08

**Authors:** Jonathan D. Baghdadi, Mia Wessel, Erik R. Dubberke, Alison Lydecker, Kimberly C. Claeys, Carolyn Alonso, K.C. Coffey, Michael Durkin, Anne J. Gonzales-Luna, Alice Y. Guh, Jennie H. Kwon, Elise Martin, Preeti Mehrotra, Christopher R. Polage, Michael S. Pulia, Clare Rock, Andrew M. Skinner, Valerie M. Vaughn, Tara Vijayan, Michael E. Yarrington, Daniel J. Morgan, for the CDC Prevention Epicenters Program

**Affiliations:** 1 University of Maryland, Baltimore, Baltimore, MD, USA; 2 Washington University School of Medicine, St. Louis, MO, USA; 3 Beth Israel Deaconess Medical Center, Boston, MA, USA; 4 University of Houston College of Pharmacy, Houston, TX, USA; 5 Centers for Disease Control and Prevention, Atlanta, GA, USA; 6 University of Pittsburgh School of Medicine, Pittsburgh, PA, USA; 7 Duke University School of Medicine, Durham, NC, USA; 8 University of Wisconsin School of Medicine and Public Health, Madison, WI, USA; 9 Johns Hopkins University School of Medicine, Baltimore, MD, USA; 10 University of Utah School of Medicine, Salt Lake City, UT, USA; 11 UCLA School of Medicine, Los Angeles, CA, USA

## Abstract

**Background:**

*Clostridioides difficile* infection (CDI) may be misdiagnosed if testing is performed in the absence of signs or symptoms of disease. This study sought to support appropriate testing by estimating the impact of signs, symptoms, and healthcare exposures on pre-test likelihood of CDI.

**Methods:**

A panel of fifteen experts in infectious diseases participated in a modified UCLA/RAND Delphi study to estimate likelihood of CDI. Consensus, defined as agreement by >70% of panelists, was assessed via a REDCap survey. Items without consensus were discussed in a virtual meeting followed by a second survey.

**Results:**

All fifteen panelists completed both surveys (100% response rate). In the initial survey, consensus was present on 6 of 15 (40%) items related to risk of CDI. After panel discussion and clarification of questions, consensus (>70% agreement) was reached on all remaining items in the second survey. Antibiotics were identified as the primary risk factor for CDI and grouped into three categories: high-risk (likelihood ratio [LR] 7, 93% agreement among panelists in first survey), low-risk (LR 3, 87% agreement in first survey), and minimal-risk (LR 1, 71% agreement in first survey). Other major factors included new or unexplained severe diarrhea (e.g., ≥ 10 liquid bowel movements per day; LR 5, 100% agreement in second survey) and severe immunosuppression (LR 5, 87% agreement in second survey).

**Conclusion:**

Infectious disease experts concurred on the importance of signs, symptoms, and healthcare exposures for diagnosing CDI. The resulting risk estimates can be used by clinicians to optimize CDI testing and treatment.

## Background

*Clostridioides difficile* is the most common cause of infectious healthcare-associated diarrhea.^1,2^ In recent years, highly sensitive nucleic acid amplification tests (NAATs) for toxigenic strains, either alone or as part of a multistep testing algorithm, have become a common method for the diagnosis of *C. difficile* infection (CDI).^3^ However, because of their high analytical sensitivity and lack of assessment of toxin production, NAATs cannot distinguish between colonization and infection. Misdiagnosis can occur when test results are misinterpreted or falsely positive in the setting of inappropriate testing.^4,5^ To support accurate diagnosis of CDI, clinicians should determine whether to test and how to interpret the results based on the individual patient’s risk.^6^

Clinicians in practice tend to overestimate the likelihood of bacterial infection.^7^ When misdiagnosis of CDI leads to unnecessary treatment, potential harms to the patient include gut dysbiosis, colonization and infection with antibiotic-resistant organisms such as vancomycin-resistant enterococci, and exposure to the high cost-share of preferred therapies.^8–10^ Paradoxically, misdiagnosis of CDI can increase risk of subsequent CDI.^10^ Even when not associated with harm from unnecessary treatment, misdiagnosis can cause harm by leading to premature closure of the differential diagnosis, delaying identification of the true cause of the patient’s symptoms.^11^ Misdiagnosis of CDI also has a negative impact on healthcare facilities, since these events are typically reported publicly as infections.^12,13^

Though sophisticated models have been developed to predict *C. difficile* test positivity,^14^ they are nonetheless dependent on the clinician’s decision to test. The purpose of this study was to inform the decision to test and subsequently diagnose CDI by developing consensus on how signs, symptoms, and healthcare exposures influence the likelihood of disease. Consensus estimates were used to build a simple, transparent, and publicly available diagnosis calculator to guide estimation of pre- and post-test probability of CDI.

## Materials and methods

We used a modified version of the RAND/UCLA Delphi Method to develop expert consensus on how signs, symptoms, and healthcare exposures influence the likelihood of primary CDI among adult inpatients, outpatients, and residents of skilled nursing facilities.^15^ This methodology systematically and quantitatively combines expert opinion with evidence when published data are insufficient.^16^ Primary CDI was defined as the first episode of CDI experienced by a given patient. The focus of this study was diagnosis of primary CDI; thus, risk factors associated with recurrent CDI or severe CDI were not addressed.

Initial estimates of the incidence of primary CDI in patient populations and on the influence of signs, symptoms, and healthcare exposures on likelihood of primary CDI were informed by a review of the literature by lead investigators (DJM, EDK, JDB). Relative influence was expressed as a likelihood ratio (LR) that modifies the pre- and post- test probability of primary CDI. Generally, an LR >5 was considered a strong effect, LR 3–5 was considered moderate, and LR 1.5–3 was considered weak.^11,12^

A group of 15 clinicians with expertise related to *C. difficile* were invited to participate as expert panelists. Expertise was determined by peer recognition for the provision of patient care, clinical microbiology, public health epidemiology, infection control, medical education, and/or research. Panelists included individuals working at federal agencies, academic medical centers, community hospitals, cancer and solid organ transplant centers, outpatient clinics, and skilled nursing facilities. Panelists were currently located in 10 states, including Missouri (3), Maryland (2), North Carolina (2), Massachusetts (2), Pennsylvania, Georgia, Texas, Wisconsin, Utah, and California.

Panelists were asked to assess the accuracy of initial estimates of incidence and risk in a survey administered via REDCap. The survey included a link to the literature review used as the basis for all estimates. The pre-defined threshold for consensus on each survey item was >70% agreement that the estimate was appropriate.^17^ Once the initial survey was completed by all panelists, a virtual Zoom meeting was scheduled to discuss items without consensus. During the session, panelists were invited to provide additional resources or relevant citations not included in the initial literature review. After the session, survey questions covering topics without consensus were clarified based on panelist input, and a follow-up survey including only those questions was distributed. This study was reviewed and determined to be exempt by the Institutional Review Board of the University of Maryland School of Medicine.

## Results

All fifteen panelists completed the initial survey, and consensus was found on 6 of 15 eligible items (40%). Fourteen panelists attended the subsequent Zoom meeting to discuss items without consensus. The remaining panelists reviewed notes from the meeting and provided feedback later over e-mail. All fifteen panelists then completed the second survey, and consensus was found on the remaining 9 items (Table 1).

**Table 1.** Consensus estimates of incidence rates of and risk factors for primary *C. difficile* infection

	Initial Estimate	Panel Agreement	Discussion points	Revised Estimate	Panel Agreement	Final Result
**Incidence Rate**						
Hospital inpatients	1% per year	40%	• Official estimates from NHSN and EIP may reflect overdiagnosis with NAAT • Some who test positive are receiving laxatives or have an alternative cause for symptoms	**1%**	87%	Consensus
SNF Residents	1% per year	40%	• Depends on definition of long-term care (category refined to “skilled nursing facility”) • In clinical experience, patients referred from SNF frequently test positive • In published literature, incidence is < 1% • SNFs may be more likely to test their patients and send to the ED in the setting of diarrhea	**2%**	87%	Consensus
Outpatients	0.05% per year	60%	• Outpatients may have healthcare exposures • Incidence in the community is increasing • True community-acquired CDI without healthcare exposure may occur but is rare	**0.05%**	93%	Consensus
**Risk Factors** ^*^						
High-risk antibiotics	**LR 7**	93%	--	--	--	Consensus
Low-risk antibiotics	**LR 3**	87%	--	--	--	Consensus
Proton pump inhibitor	**LR 2**	87%	--	--	--	Consensus
Severe immunosuppression^**^	LR 3	53%	• Complicated exposure that may have differential effects on colonization, infection, and severity of illness • May be difficult to define, and heterogeneity exists between patients based on medications and underlying conditions • Frequently confounded by other factors • Severely immunosuppressed patients may be more likely to undergo testing for subtle or minor symptoms	**LR 5**	87%	Consensus
Non-severe immunosuppression^†^	**LR 1.5**	73%	--	--	--	Consensus
Hospitalization in prior 60 days	LR 1.5	53%	• Hospitalization frequently involves antibiotics • Antibiotic exposure is the main risk factor	**LR 2**	100%	Consensus
New/unexplained Severe Diarrhea^††^	LR 3	33%	• Symptoms need to be assessed in context (e.g., severe diarrhea does not increase risk if an alternative cause is already known) • Symptoms differentiate colonization and infection • Should be distinguished from toxic megacolon	**LR 5**	100%	Consensus
New severe leukocytosis (e.g., >20k)	LR 1.5	60%	• Interpretation depends on the patient population • Leukocytosis is relevant when new and severe	**LR 3**	93%	Consensus
Fever	LR 1.5	60%	• Not a significant risk factor	**LR 1**	93%	Consensus
Minimal Symptoms	**LR 0.2**	87%	--	--	--	

LR, likelihood ratio; NHSN, National Healthcare Safety Network; EIP, Emerging Infections Program; NAAT, nucleic acid amplification testing; SNF, skilled nursing facility.

Consensus values for incidence rate and likelihood ratio have been bolded.

* Through discussion with the expert panel, consensus was reached that the appropriate window period for medication and healthcare exposures was 60 days.

** Severe immunosuppression was defined as hematologic malignancy, status-post stem cell transplant, or recent solid organ transplant.

† Non-severe immunosuppression was defined as medications used to treat patients with non-hematologic malignancy or remote solid organ transplant.

†† E.g., ≥ 10 liquid bowel movements per day.

### Incidence - hospital inpatients

In the first-round survey, consensus was not reached on the incidence of CDI in any of the queried populations of interest. Based on review of the literature, the incidence of CDI among hospital inpatients was estimated to be approximately 1% in 1 year (see Table 1).^1,18,19^ In the first round of surveys, the expert panel was divided on this estimate, with 6 (40% of n = 15) agreeing that 1% was appropriate, 5 (33%) estimating that the true incidence was lower, and 4 (27%) estimating that the true incidence was higher. In discussion, panelists noted that overdiagnosis may inflate published estimates of incidence. Thus, for the second-round survey, we did not change the estimated incidence among hospital inpatients from 1%. However, based on discussion during the meeting, the panel now reached consensus with 13 (87%) experts agreeing that this estimate was appropriate.

### Incidence - residents of skilled nursing facilities

Initially, the incidence of CDI among residents of skilled nursing facilities (SNFs) was estimated to be equivalent to the incidence among hospital inpatients (1% in 1 year).^20^ In the first-round survey, the panel was split on this estimate, with 6 (40%) agreeing that 1% is appropriate, 2 (13%) perceiving the true incidence as lower than 1%, and 7 (47%) perceiving the true incidence as higher than 1%. Discussion during the meeting focused on tension between the incidence in published data, which is lower than 1%,^20^ and the incidence in the panel’s clinical experience, which seems higher than 1%. Panelists noted that SNF residents experience similar exposures to healthcare as do hospital inpatients, and they debated if residence in a SNF itself confers risk independent of other factors. The panel also suggested that reported estimates of CDI in this population may be more accurate than in other populations, since SNF residents may be more readily tested at the onset of symptoms. Based on this discussion, the estimated incidence was increased to 2% for the second-round survey. Consensus was reached on this new estimate, with 13 (87%) panelists in agreement.

### Incidence - outpatients

The initial estimate of incidence among outpatients was 0.05%.^21^ In the first-round survey, 9 (60%) panelists agreed with this estimate, 2 (13%) perceived the true incidence as lower than 0.05%, and 4 (27%) perceived the true incidence as higher than 0.05%. Panel discussion emphasized that healthcare exposures are common in the community, though panelists noted that about 1 in 3 cases of CDI occur without apparent recent exposure to antibiotics. However, the panel determined that any potential adjustments to the estimated incidence among outpatients would be minor and likely not change pre- or post-test estimates of CDI probability substantially. In the second-round survey, the estimate was not adjusted from 0.05%. However, based on discussion, 14 (93%) expert panelists now agreed that this estimate was appropriate.

### Medication exposures—antibiotics

In the first-round survey, there was consensus within the panel that antibiotics are appropriately grouped into three categories based on risk: high-risk antibiotics, low-risk antibiotics, and minimal-risk risk antibiotics (n = 10, 71%). High-risk antibiotics were considered a strong risk factor for CDI (LR 7, n = 14, 93% of the panel), and low-risk antibiotics were considered a moderate risk factor (LR 3, n = 13, 87%). Antibiotics considered high- and low-risk by the expert panel are provided in Table 2. Regarding other medications, >70% of panelists agreed in the first-round survey that proton pump inhibitors (LR 2, n = 13, 87%) and non-severe immunosuppression (LR 1.5, n = 11, 73%) are associated with increased risk. Non-severe immunosuppression was defined by medications used to treat patients with non-hematologic malignancy or remote history of solid organ transplant.

**Table 2.** CDI risk associated with antibiotic exposure based on consensus from expert panel

High Risk (LR 7)	Low Risk (LR 3)	Minimal or Negligible Risk (LR 1)
• Clindamycin^*^ • Fluoroquinolones^*^ • 3^rd^/4^th^ generation cephalosporins^*^ • Carbapenems • Piperacillin/tazobactam and other beta-lactam + beta-lactamase inhibitors • Oral/enteral vancomycin	• 1^st^/2^nd^ generation cephalosporins • Beta-lactams without beta-lactamase inhibitors, including aminopenicillins and anti-staphylococcal penicillins • Macrolides	• Tetracyclines • Intravenous vancomycin • Trimethoprim/sulfamethoxazole • Metronidazole • Nitrofurantoin • Fosfomycin

CDI, *C. difficile* infection; LR, likelihood ratio associated with diagnosis of CDI.

Antibiotics were placed into risk categories based on suggestions from the expert panel. Only antibiotics (or antibiotic classes) receiving at least 2 votes from the expert panel are included in this table. Antibiotics that were suggested for more than one category are listed in the category where they received the most votes.

* Antibiotics receiving votes from >10 panelists (indicating strong agreement).

The period of time at-risk for CDI after an antibiotic exposure was initially estimated to be 8 weeks.^22,23^ In the first-round survey, 8 panelists (53%) agreed with this estimate, 2 (13%) perceived 8 weeks as too long, and 5 (33%) perceived 8 weeks as too short. Panel discussion acknowledged that risk of CDI after antibiotic exposure is better represented by a spectrum than a precise value. Risk may persist for 6 months or longer but generally decreases with time. One panelist noted that the gut microbiome of most healthy adults will return to normal within 2 months of antibiotic exposure.^24^ For the second-round survey, the antibiotic exposure window was adjusted to 60 days, which was suggested by the panel as a reasonable cut-off. Fifteen of 15 (100%) panelists agreed that this estimate was appropriate.

### Medication exposures—severe immunosuppression

Review of the literature demonstrated substantial heterogeneity in the risk of CDI associated with severely immunosuppressed patients, defined as either patients on active treatment for hematologic malignancy or recent solid organ transplant.^25–27^ The initial estimate of risk was an LR of 3. In the first-round survey, 8 (53%) panelists agreed with this estimate, 1 (7%) panelist perceived LR 3 as too high, and 6 (40%) perceived LR 3 as too low. Discussion within the panel emphasized that severe immunosuppression is a complicated risk factor that may be difficult to disentangle from other healthcare exposures. One panelist noted that severely immunosuppressed patients may be more likely to undergo testing, inflating estimates of diagnosis in this population. For the second-round survey, the risk estimate for severe immunosuppression was adjusted to LR 5. Thirteen (87%) panelists agreed that this estimate is appropriate, and 2 (13%) perceived this estimate as too high.

### Other exposures - recent hospitalization

The initial risk estimate for CDI after recent hospitalization was LR 1.5.^23^ “Recent” was defined as within the previous 60 days, which was the same exposure window as for antibiotics. In the first-round survey, 8 (53%) panelists agreed with this estimate and 7 (47%) perceived it as too low. Discussion within the panel acknowledged the difficulty of assessing hospitalization as an independent risk factor since hospitalized patients are frequently exposed to antibiotics. In the second-round survey, the risk estimate was adjusted to LR 2. All panelists agreed that this estimate was appropriate.

### Signs and symptoms – minimal or no diarrhea

Risk estimates for CDI associated with specific signs and symptoms were defined based on expert consensus. Thirteen (87%) panelists agreed that when testing is performed in the setting of minimal symptoms, the likelihood of CDI is substantially decreased (LR 0.2). During the panel meeting, it was noted that overtesting remains an issue among minimally symptomatic patients.

### Signs and symptoms – severe diarrhea

“Clinically significant” diarrhea was defined as 3 liquid stools in a 24-hour period. Initially, severe diarrhea far exceeding this threshold (for example, >10 liquid bowel movements per day) was estimated to increase risk of CDI by LR 3. In the first-round survey, 5 (33%) panelists agreed with this risk estimate, and 10 (67%) perceived it as too low. Panel discussion focused on the importance of assessing symptoms in context, including specifically if an alternative cause of diarrhea is already known. The panel also suggested that patients with ileus or toxic megacolon should be placed in a separate category. In the second-round survey, “severe diarrhea” was rephrased as “new or unexplained severe diarrhea,” and the risk estimate was increased to LR 5. Fifteen of 15 (100%) panelists agreed with this estimate.

### Signs and symptoms – fever

Initially, fever was estimated to increase the likelihood of a diagnosis of CDI by LR 1.5. In the first-round survey, 9 (60%) panelists agreed with this estimate, 5 (33%) perceived LR 1.5 as too high, and 1 (7%) perceived LR 1.5 as too low. During discussion, the panel suggested de-emphasizing the importance of fever in the diagnosis of CDI, with multiple panelists suggesting fever was not a significant risk factor. One panelist noted that fever be assigned an LR close to 1, allowing its minimal impact to stand in contrast to the larger LRs assigned to other risk factors. For the second-round survey, the LR for fever was adjusted to 1.1. Fourteen (93%) panelists agreed that this estimate was appropriate, and 1 (7%) perceived it as too low.

### Signs and symptoms—leukocytosis

The initial risk estimate was that leukocytosis was equivalent to fever (LR 1.5). In the first-round survey, 9 (60%) panelists agreed with this estimate, 1 (7%) perceived LR 1.5 as too high, and 5 (33%) perceived LR 1.5 as too low. During discussion, leukocytosis was perceived as being more important for CDI diagnosis than fever, particularly when the white blood cell count is very high. Mild or mid-range leukocytosis was perceived as not impacting the diagnosis of CDI. In the second-round survey, “leukocytosis” was rephrased as “new severe leukocytosis (e.g., >20k)” and estimated to increase risk by LR 3. Fourteen panelists (93%) agreed that this risk estimate was appropriate, and 1 (7%) perceived it as too high.

## Discussion

In this study, we synthesized the literature with clinical judgment from an expert panel to quantify the impact of signs, symptoms, and healthcare exposures on the diagnosis of CDI. Though members of the panel emphasized the difficulty of generating simple risk estimates when considering the heterogeneity among patients with CDI, the panel found consensus on all items discussed after one round of discussion and two surveys. Consensus risk estimates were then used to develop a diagnosis calculator to guide testing ordering and subsequent diagnosis of CDI (https://calculator.testingwisely.com/).

The strengths of our consensus risk estimates and the diagnosis calculator they were used to build are that they are accessible, transparent, and teachable. Because our risk model is simple and relatively easy to understand, it can be used on rounds or in the classroom to guide assessment and discussion of an individual patient’s risk of CDI without cost, programming time, or troves of clinical data. More sophisticated models, including models developed using machine learning or other techniques based on data science, are likely more accurate on a case-by-case basis but too opaque and hyper-specific to the training data to offer generalizable insights.^14^ Further, our risk model was developed with the explicit purpose of informing when a CDI should be appropriately performed. Sophisticated models that predict when a test is positive may be biased if they do not exclude instances of inappropriate testing.

Healthcare exposures that increase risk of CDI, including antibiotics, immunosuppression, and inpatient environments, tend to cluster together within certain patient populations.^23^ When these patients develop diarrhea, the pre-test likelihood of CDI can be very high. For most clinicians, these patients represent the “illness script” for CDI that is most salient to testing and treatment decisions. However, up to 50% of CDI cases are community-associated, and a subset may occur in patients without risk factors.^1,21,28^ One of the goals of this project was to understand the risk of CDI when a patient’s presentation does not match the typical illness script.

The consensus estimates of risk from this study should be considered associations that do not imply causal relationships. For instance, we found consensus that severe immunosuppression is associated with moderate risk of primary CDI based on the published literature and the clinical experience of our panelists. However, it is uncertain whether immunosuppression significantly increases risk of CDI in the absence of antibiotics to induce gut dysbiosis. Because risk factors tend to cluster within patients, disentangling the impact of a single risk factor was considered too difficult.

A key function of our diagnosis calculator is to help distinguish between cases of colonization and infection due to *C. difficile* using clinical data. However, at the population level, these distinctions are not always clear. Among the items addressed in this study, estimates of CDI incidence across patient populations provoked the most disagreement among experts. The CDC publishes detailed annual reports on the incidence and outcomes of CDI at 10 sites across the US through its Emerging Infections Program (EIP). In 2020, 31% of CDI cases reported by the EIP included a positive NAAT and negative toxin assay. Some of these cases may represent colonization, while others are likely true infections associated with a false-negative toxin assay. Though cases of colonization inflate estimates of CDI incidence, underreporting may occur when patients are diagnosed with or treated empirically for CDI in the absence of a positive test result. Though NAATs are highly sensitive, false negatives do occur,^29^ and clinicians may treat without testing as a workaround for clinical decision support or if otherwise disincentivized to report cases.^30^ If the local incidence of CDI is known, this value can be entered manually into the diagnosis calculator to provide a more accurate baseline for risk estimates.

It is notable that our expert panel perceived fever as inconsequential to the diagnosis of CDI. CDI testing is included as a standard component of the “fever work-up” in some practice settings, even when diarrhea is not present.^31,32^ Our experts perceived this practice as inappropriate, intentionally contrasting its lack of diagnostic value with other features of disease. To put things in perspective, based on the assigned LR of 1.1, a patient with a pre-test probability of 10% for CDI who is found to be febrile would have their probability adjusted to 11%. The same patient, if afebrile but found to have a severe leukocytosis, would have their probability of CDI increased to 25%.

### Limitations

Our study synthesized the published literature with expert opinion to support the diagnosis of CDI. However, we did not conduct a systematic review and instead relied on our participating experts to identify relevant data. Expert opinion was elicited via surveys, during a virtual Zoom meeting, and over e-mail. During the Zoom meeting, we encouraged an open discussion in which all could participate, but individuals with dissenting opinions may have felt less comfortable sharing than if we had conducted individual interviews. Further, estimated LRs cannot account for differences in the level of risk that may occur based on the intensity of healthcare exposures, and estimates of incidence do not consider regional, local, or unit-level variation that may develop due to specific risk factors, inadequate infection control practices, or outbreaks. It is beyond the scope of this study to consider how our risk estimates or diagnosis calculator might be implemented clinically, but we suspect their value lies in educating trainees and informing discussions within the healthcare team on the use and interpretation of testing for CDI. Finally, our risk model focused on signs, symptoms, and healthcare exposures, rather than baseline health status or comorbidities. This focus meant that important risk factors, including age and inflammatory bowel disease, were not included. However, we note that the relationship between risk factors is complicated, and the impact of age is less significant after accounting for risk from other factors.^33^

## Conclusions

We synthesized published evidence with expert opinion to support accurate diagnosis of CDI. The resulting risk estimates can be used by clinicians, either on their own or in the context of an online calculator, to optimize testing and treatment for patients with suspected CDI.
